# Vulnerability of Store-Operated Calcium Entry to Inhibitors and Microenvironment in Cells of Different Breast Cancer Subtypes

**DOI:** 10.3390/life14030357

**Published:** 2024-03-08

**Authors:** Anton Y. Skopin, Lubov N. Glushankova, Konstantin O. Gusev, Elena V. Kaznacheyeva

**Affiliations:** Institute of Cytology RAS, Tikhoretsky Ave. 4, 194064 St. Petersburg, Russia

**Keywords:** breast cancer, microenvironment, hypoxia, store-operated calcium entry, Leflunomide, Teriflunomide, Auxora, BTP2

## Abstract

The incidence and development of cancer are highly dependent on pathological disturbances in calcium homeostasis of the cell. One of the major pathways for calcium entry is store-operated calcium entry (SOCE), which functions in virtually all cell types. Changes in the expression level of the main proteins organizing SOCE are observed during the development of various cancer types, particularly breast cancer (BC). This leads to unique SOCE with characteristics individual for each type of BC and requires particular therapeutic approaches. In this study, we tested the sensitivity of SOCE in various BC cells to selective ORAI channel inhibitors and the less selective compounds Leflunomide and Teriflunomide, approved by the FDA for clinical use. We also analyzed the vulnerability of SOCE to the influence of factors typical of the tumor microenvironment: hypoxia and acidification. We have observed that the SOCE inhibitors Leflunomide and Teriflunomide suppress SOCE in the triple-negative BC cell line MDA-MB-231, but not in the luminal A BC cell line MCF-7. MDA-MB-231 cells also demonstrate higher pH dependence of SOCE compared to MCF-7 cells. In addition, the oxygen scavenger sodium dithionide also affects SOCE, stimulating it in MDA-MB-231 cells but inhibiting in MCF-7 cells. Overall, our data highlight the importance of considering the different sensitivities of various BC cell types to inhibitors and to microenvironmental factors such as hypoxia and acidification when developing targeted drugs.

## 1. Introduction

Calcium triggers and regulates various cellular functions such as secretion, proliferation and migration. Ca^2+^ signaling is also a key signal transducer in the intrinsic apoptosis pathway. In cancer cells, it determines the occurrence and further development of tumors [1].

One of the major pathways for calcium entry into nonexcitable cells is store-operated calcium channels (SOCs), located in the plasma membrane and activated in response to Ca^2+^ depletion in the endoplasmic reticulum (ER). Their functioning is necessary both for replenishing Ca^2+^ in the ER and for transmitting a large number of intracellular signals [2,3]. The main molecular components of store-operated calcium entry (SOCE) are the STIM and ORAI proteins, as well as some members of the TRPC family [4,5,6,7].

SOCE organizing proteins have been found to be involved in pathological processes in various types of cancer, including leukemia, prostate cancer, non-small cell lung adenocarcinoma and breast cancer (BC). So, in the majority of transformed cells, the expression levels of STIM, Orai and TRPC proteins are increased relative to cells of a non-oncogenic nature, which impacts the ratio of the proteins STIM1 and STIM2, and Orai1, Orai2 and Orai3 [8,9,10,11,12,13]. This leads to a specific organization of SOCE for each cell line and, therefore, to specific consequences caused by a unique Ca^2+^ signal.

This entails the need for the individual selection of drugs aimed at normalizing the SOCE for each type of cancer, which is necessary for successful cancer therapy [14].

In particular, some authors proposed the Orai3 channel as a target for BC therapy [10,15]. However, no selective modulators of Orai3 activity have yet been identified. Currently, effective and selective inhibitors have been found only for the Orai1 and Orai2 channels [16]. We recently found a promising inhibitor of the STIM2-dependent signaling pathway, which offers an alternative pathway for normalizing SOCE [17].

Interestingly, modulators of SOCE can also be found among drugs originally intended to treat other pathologies. For example, the drugs Leflunomide (Lef) and Teriflunomide (Ter) are approved for the treatment of autoimmune diseases as inhibitors of dihydroorotate dehydrogenase, but at the same time, they also demonstrate an inhibitory effect on SOCE [18]. Currently, these compounds are considered non-selective antiproliferation agents and are undergoing clinical trials as potential anticancer drugs, including the treatment of BC [19,20,21]. The effect of Leflunomide and Teriflunomide on SOCE in cancer cells has not been sufficiently studied. Understanding their influence on SOCE with different protein compositions may allow us to develop more specific therapeutic approaches, particularly for BC treatment [22].

Breast cancer is one of the most common types of cancer. There are different subtypes of BC according to the gene expression profile of endorphin receptors, progesterone receptors and human epidermal growth factor receptor 2 (HER2). Triple-negative (TNBC) subtypes are negative for all three receptors and are the most aggressive forms of BC [23].

In this work, various inhibitors of SOCE were studied in two BC cell lines with polar SOCE molecular composition: (1) the TNBC line MDA-MB-231 with predominant expression of Orai1, and (2) the luminal A BC line MCF7 in which SOCE is mainly determined by Orai3 proteins [24,25]. A non-oncogenic line, MCF-10A, obtained from a patient with mastopathy, was used as a control cell line [13,26,27]. We studied the effect of the selective SOCE inhibitors CM4620 and BTP2 or non-selective FDA-approved pharmacological agents, Leflunomide and Teriflunomide, on SOCE in different BC cell lines. Additionally, we studied the influence on SOCE of tumor microenvironmental factors such as hypoxia and acidification [28,29].

## 2. Materials and Methods

### 2.1. Materials

CM4620 (Auxora) (MedHemExpress, Princeton, NJ, USA), BTP2 (Calbiochem, San Diego, CA, USA), Tg, Leflunomide, Teriflunomide (Sigma-Aldrich, Saint Louis, MO, USA), Sodium dithionite (Lenreactive, St. Petersburg, Russia), DMSO (Sigma-Aldrich, Saint Louis, MO, USA).

### 2.2. Cell Culture

MCF-7 cells (Institute of Cytology RAS, St. Petersburg, Russia) were cultured in an EMEM medium (Biolot, St. Petersburg, Russia) supplemented with 10% fetal bovine serum (HyClone, Logan, UT, USA), 1% NEAA (ICN Biomedicals, Santa Ana, CA, USA) and 10 μg/mL Insulin (PanEco, Moscow, Russia).

MCF-10A cells (ATCC) were cultured in a DMEM/F12 medium (Biolot, Russia) supplemented with 5% fetal horse serum (Sigma-Aldrich, USA), 20 ng/mL EGF (Institute of Cytology RAS, Russia), 10 μg/mL Insulin (PanEco, Russia), 0.5 μg/mL Hydrocortisone and 1 μg/mL Isoproterenol (Sigma-Aldrich, USA).

MDA-MB-231 cells (ATCC) were cultured in a DMEM medium (Biolot, Russia) supplemented with 10% fetal bovine serum (HyClone, USA).

All cell lines were cultured in the presence of penicillin (100 U/mL) and streptomycin (0.1 mg/mL) at 37 °C and 5% CO_2_.

### 2.3. Fluorescence Measurements

Changes in the intracellular Ca^2+^ concentration were measured using a Fura-2 AM calcium indicator (Thermo Fisher Scientific, Waltham, MA, USA). The cells were plated into 96-well culture plates (Jet Biofil, Elgin, IL, USA) 48 h prior to analysis. HBSS solution contained (in mM): 2 CaCl_2_, 130 NaCl, 2.5 KCl, 1.2 MgCl_2_, 10 glucose, 10 HEPES (pH 7.4). The cells were incubated in an HBSS solution containing 5 μM Fura-2 AM (Invitrogen, Waltham, MA, USA) for 40 min and washed out for a further 30 min in HBSS at room temperature. Measurements were performed using a Varioskan LUX reader (Thermo Fisher Scientific, USA).

### 2.4. Statistics

Data were analyzed with Excel 2019 (Microsoft) and OriginPro 2018 (OriginLab). The significance of the differences between mean values was determined by the Mann–Whitney U test. *p* values < 0.05 were considered significant. The number of replicates was 9–12. Data are presented as mean ± SEM.

## 3. Results

### 3.1. Effect of Selective SOCE Inhibitors on Breast Cancer Cells

To characterize the Ca^2+^ response of cells, we used a calcium imaging technique with a Fura-2 indicator. To deplete Ca^2+^ stores and activate SOCE, thapsigargin (Tg), a SERCA blocker, was applied in the presence of extracellular Ca^2+^. The obtained Ca^2+^ response reflected the sum of Ca^2+^ release and the subsequent Ca^2+^ entry. The SOCE amplitude was analyzed at the ninth minute after Tg application, since the influence of the Ca^2+^ release at this moment is minimal.

**Figure 1:**
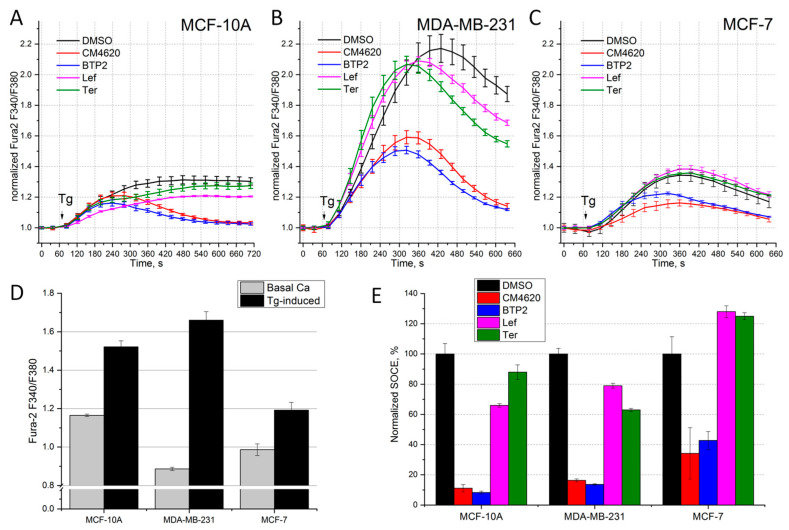
Effect of SOCE inhibitors in BC cells. The Tg-induced Ca^2+^ response after 25–35 min incubation with 5 µM CM4620 (red), 5 µM BTP2 (blue), 50 µM Leflunomide (Lef, pink), 50 µM Teriflunomide (Ter, green) or 0.5% DMSO (control, black) in cell lines MCF-10A (**A**), MDA-MB-231 (**B**), MCF-7 (**C**). The arrow indicates the time of 1 μM Tg application. Shown is the ratio of Fura-2 fluorescence at 340 and 380 nm, normalized to basal calcium level over time (n = 9–12). (**D**) Ca^2+^ level at rest and at the 9th minute after Tg application in cell lines MCF-10A, MDA-MB-231, MCF-7. Shown is the ratio of Fura-2 fluorescence at 340 and 380 nm. (**E**) Amplitude of the Ca^2+^ response at the 9th minute after Tg application in cell lines MCF-10A, MDA-MB-231, MCF-7, incubated with 5 μM CM4620 (red), 5 μM BTP2 (blue) normalized to the Ca^2+^ amplitude measured in presence of 0.5% DMSO (control, black). The mean and SEM are shown.

The studied cell lines demonstrated different Ca^2+^ responses induced by Ca^2+^ store depletion caused by the application of 1 μM Tg (Figure 1A–C). So, in the non-oncogenic cell line MCF-10A, used as a control, the calcium at the ninth minute increased by 30 ± 2.4%, while in the MDA-MB-231 cell line, the Ca^2+^ increased by 87 ± 5% above the basal level (Figure 1A,B,D). In the MCF-7 cell line, the Ca^2+^ increased by 20 ± 4%, which is the lowest response among the cell lines studied (Figure 1C,D).

To elucidate the contribution of the channels formed by the Orai1 and Orai2 proteins to the observed Ca^2+^ response of the cells to Ca^2+^ store depletion, we used CM4620, a selective inhibitor of Orai channels (IC50 = 119 nM for Orai1 and 850 nM for Orai2) [30]. In addition, for the inhibitory analysis of SOCE, we used a blocker, BTP2, the target of which is considered to be channels formed by Orai proteins [31]. Figure 1E shows the SOCE amplitude values at the ninth minute after 1 μM Tg application in the presence of the indicated inhibitors, normalized relative to the control values. In all three cell lines, incubation for 25–35 min with the compounds CM4620 or BTP2 significantly blocked SOCE, the maximum suppression of the Ca^2+^ response (by 89% and 92%, respectively) was recorded in the MCF-10A cell line, and the minimum suppression was observed in the MCF-7 cells (by 68% and 65%, respectively). In the MDA-MB-231 cell line, CM4620 and BTP2 suppressed SOCE by 84% and 86%, respectively (Figure 1, Table 1). Thus, BTP2 and CM4620 showed similar suppression with different efficiencies when comparing the cell lines with each other.

### 3.2. Effect of Nonspecific SOCE Blockers Leflunomide and Teriflunomide

Leflunomide and Teriflunomide have similar chemical structures. These compounds block the mitochondrial enzyme dihydroorotate dehydrogenase, have an inhibitory effect on some tyrosine kinases and the transcription factor NF-kB, as well as SOCE [18,19,21].

Figure 1 shows the Tg-induced Ca^2+^ responses after 25–35 min incubation of cells with 50 μM Lef or 50 μM Ter. The maximum suppression of the Ca^2+^ response in the presence of Leflunomide was recorded in the MCF-10A cell line (Figure 1A). Lef caused a reduction in SOCE by 34%, while Ter caused only a 12% reduction (Figure 1E). In contrast, in the MDA-MB-231 cells, Ter caused a greater reduction in the SOCE compared to Lef, by 36% and 21%, respectively (Figure 1B,D, Table 1). In the MCF-7 cell line, which is known to overexpress Orai3 protein, Lef and Ter did not affect the calcium response, which may reflect that Orai3 is not sensitive to these blockers (Figure 1C,E, Table 1). Thus, the less selective inhibitors Lef and Ter disrupt Ca^2+^ signaling in cells of the most aggressive TNBC, but do not affect SOCE in the ER-positive subtype of BC.

### 3.3. Sensitivity of SOCE in Breast Cancer Cells to the Extracellular pH

**Figure 2:**
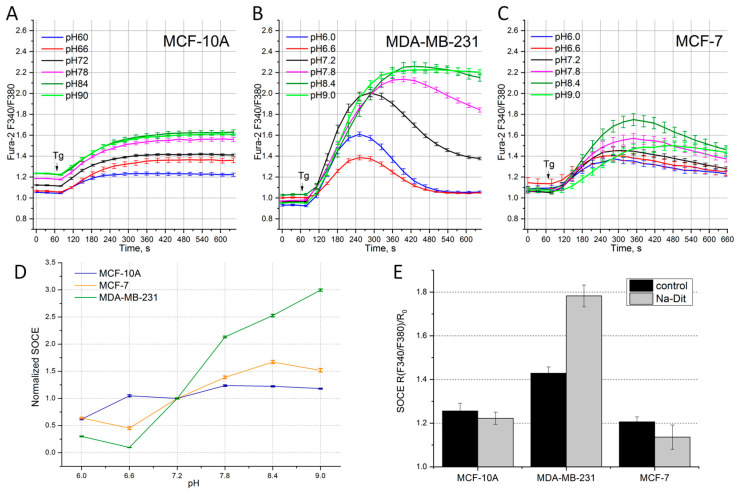
Effect of extracellular pH and hypoxia in different BC cell lines. Tg-induced SOCE measured at various pH levels in BC cell lines MCF-10A (**A**), MDA-MB-231 (**B**), MCF-7 (**C**). The arrow indicates the time of 1 μM Tg application. Cells were preincubated for 25–45 min in solutions with various pH levels. Shown is the ratio of Fura-2 fluorescence at 340 and 380 nm as a function of time. (**D**) Amplitude of Ca^2+^ response, measured at the 9th minute, normalized to basal calcium level, relative to amplitude at external pH 7.2 in lines MCF-10A, MDA-MB-231, MCF-7. (**E**) Amplitude of Ca^2+^ response, measured at the 9th minute, normalized to basal calcium level in cells MCF-10A, MDA-MB-231, MCF-7 incubated for 25 min in solutions with 1 mM Na-Dit. Shown are the means and SEMs (n = 9–12).

In addition, we studied the effect of pH on the amplitude of the Tg-induced Ca^2+^ response in different BC cell lines. The following extracellular solution pH values were used: 6.0, 6.6, 7.2, 7.8, 8.4 and 9.0. Despite the general tendency for all the cell lines to reduce the amplitude of the Tg-induced Ca^2+^ response with decreasing pH, the cell lines showed different sensitivities to changes in the extracellular pH (Figure 2A–D).

In the MDA-MB-231 cells, the basal Ca^2+^ level showed minor changes with varying pH, and the amplitude of the Ca^2+^ response at the ninth minute after Tg application had a pronounced dependence on the extracellular pH level (Figure 2B). So, an increase in the pH led to a significant augmentation in the SOCE amplitude (the ratio of the maximum to minimum amplitude is 9.6) (Figure 2D). It is known that SOCE in this cell line is mainly due to the functioning of the Stim1 and Orai1 proteins [24].

Our findings assume that SOCE of this molecular composition has increased sensitivity to the extracellular pH.

In the MCF-7 cell line, with increased expression of Orai3, the basal Ca^2+^ level was practically independent of the extracellular pH (Figure 2C). However, unlike the MDA-MB-231 cells, in the MCF-7 line, the dependence of SOCE on an increase in the pH was less pronounced, while maintaining an increasing trend (Figure 2D). The ratio of the maximum to minimum amplitude for the MCF-7 cells was 3.7. Minimal changes in the calcium response depending on the external pH were recorded in the non-oncogenic cell line MCF-10A (Figure 2A). A change in the extracellular pH from alkaline to acidic caused a decrease in the basal calcium levels, and the amplitude of the Ca^2+^ response changed insignificantly (the ratio of the maximum to minimum amplitude was 2.0) (Table 2). Considering that in most cases, the pH inside the tumor shifts to acidic values, this means that the decrease in SOCE would reduce the Ca^2+^ concentration inside the tumor cells, increasing their resistance to Ca^2+^-mediated apoptosis.

### 3.4. Sensitivity of SOCE in Breast Cancer Cells to Na-Dithionite

To model hypoxic conditions, Na-dithionite (Na-Dit) was used, which is a powerful O_2_ absorber that significantly reduces the concentration of molecular oxygen. The addition of 1 mM Na-Dit to the extracellular solution causes hypoxia, characteristic of cells located inside the tumor [32,33]. A 30 min incubation with 1 mM Na-Dit had different effects on the Tg-induced Ca^2+^ response in the tested cell lines (Figure 2D, Table 2). In the MCF-10A cells, the SOCE amplitude under hypoxia was reduced by 13% relative to the control conditions. In the MDA-MB-231 cell line treated with 1 mM Na-Dit, the amplitude of SOCE is 83% greater relative to the SOCE level in the control conditions (Figure 2D, Table 2). In the MCF-7 cells, treatment with Na-Dit led to a significant decrease in the SOCE amplitude of 44% relative to the control conditions. It can be assumed that cell resistance to Ca^2+^ overload under Na-Dit-induced hypoxia depends on the amount of Orai3 in the overall SOCE structure.

## 4. Discussion

SOCE is involved in proliferation, migration and metastasis processes, which define the malignant characteristics of cancer. To perform these functions in a constantly changing microenvironment, cancer cells change the expression of certain proteins participating in SOCE, helping them adapt to external factors. In our study, we selected BC cell lines with substantially diverse SOC compositions.

We explored the effect of several external factors and inhibitory compounds on SOCE in three cell lines: the MCF-10A control cell line of a non-oncogenic nature, the HER2-negative ER-positive BC cell line MCF-7 with increased expression of Orai3 channels, and the TNBC MDA-MB-231 cell line with increased expression of Orai1 channels.

The compound BTP2 has been used for a long time as an SOCE blocker, without distinguishing between isoforms of the Orai or STIM proteins [31,34]. Later, in an overexpressed system, it was shown that BTP2 essentially abrogates ORAI1 and ORAI2 activity while causing only a partial inhibition of ORAI3 [16]. Compound CM4620 demonstrated inhibitory effects on Orai1 and Orai2, but not Orai3 [30].

Our inhibitory analysis showed that the effect of BTP2 and CM4620 on the Ca^2+^ response in each cell line studied is almost identical (Figure 1, Table 3). However, comparing cell lines with each other, we observed the different efficiencies of SOCE inhibition. The suppression of the Ca^2+^ response in the Orai3-channel-rich cell line MCF-7 was minimal among all the cell lines examined (Table 3). In these cells, BTP2 did not show greater inhibition than CM4620, suggesting that BTP2 does not influence Orai3 activity.

Leflunomide and Teriflunomide were approved by the FDA for the treatment of rheumatoid arthritis and multiple sclerosis. It is known that these compounds block the mitochondrial enzyme dihydroorotate dehydrogenase and impede the synthesis of uridine monophosphate, slowing down the synthesis of DNA and RNA [19]. Currently, several clinical trials of Leflunomide as an antiproliferative drug are being conducted for the treatment of melanoma, myeloma, glioblastoma, and metastatic TNBC [21]. Recent studies have indicated that the compounds Leflunomide and Teriflunomide inhibit SOCE [18]. However, the pharmacological mechanism of their action has not been fully studied.

In our experiments, Leflunomide and Teriflunomide showed discriminating action on SOCE in different BC cell lines. So, SOCE in the MCF-7 cells with increased Orai3 expression was not sensitive to Lef and Ter (Table 3). In contrast, Lef and Ter significantly inhibited SOCE in the MDA-MB-231 cell line (21% and 37%, respectively). SOCE in the control MCF-10A cells showed a different sensitivity to the action of the compounds (34% for Lef and 12% for Ter). Thus, Leflunomide and Teriflunomide disrupt Ca^2+^ signaling in the cells of the most aggressive BC type, TNBC, but do not affect Ca^2+^ entry in the ER-positive subtype of BC cells.

The tumor microenvironment largely determines carcinogenesis. Soluble microenvironmental factors include various cytokines, chemokines, growth factors, reactive oxygen species, secreted proteins and nutrients such as glucose [35,36,37,38,39]. In this work, we focused on microenvironmental factors such as hypoxia and changes in extracellular pH level, since these factors have a significant impact on the functioning of SOCs and the overall Ca^2+^ homeostasis in cancer cells [28,40].

The dysregulation of pH is a common characteristic of cancer cells, as they often have an increased intracellular pH and decreased extracellular pH compared to normal cells [40]. Our experiments showed that the external pH differently impacts SOCE in various BC cell lines. So, SOCE in the MDA-MB-231 cells increased by 10 times when the external pH changed from 6.6 to 9.0, while in the control non-oncogenic cell line MCF-10A, SOCE is practically non-sensitive to the variation in the pH in the external solution. MCF-7 BC cells also have a tendency to increase SOCE when external solution is alkalized. But SOCE in these cells is enhanced by only 2 times under alkalization, when the pH changes from 6.6 to 9.0 (Figure 2). These data suggest that BC cells with predominant Orai1 expression are more sensitive to pH changes compared to BC cells expressing predominantly Orai3. It is known that a low pH in the tumor environment leads to low SOCE, which allows cancer cells to escape apoptosis and to enhance proliferation and migration [41]. While MDA-MB-231 cells exhibit high SOCE at physiological conditions (Figure 1), the high sensitivity to pH changes can reflect the ability of these cells to adjust SOCE at an acidic pH for preserving typical cancer hallmark function.

Na-dithionite is a powerful O_2_ scavenger which induces hypoxia by reducing molecular O_2_. Moreover, hypoxia causes intracellular acidification of many cell types, including smooth muscle, nerve cells, cardiac cells and tumor cells, as they switch from oxidative to glycolytic metabolism [42,43]. The application of 1 mM Na-Dit to hASMC and HEK293 cells resulted in a decrease in the intracellular pH from resting levels (approximately 7.0–7.2) to a new level of 6.6–6.4, which reduces the functional coupling of STIM–Orai and decreases Ca^2+^ entry. From another side, hypoxia favors the junctional accumulation of STIM1 [32].

Interestingly, among the cell lisnes studied, the greatest inhibition (by 34%) of the Ca^2+^ response under hypoxic conditions is observed in the MCF-7 cells (Table 3), in which Orai3 plays a predominant role in SOCE. This inhibition coincides with the functional uncoupling of STIM–Orai. On the other hand, it is known that long-term hypoxia causes increased expression of the Orai3 protein [11]. Thus, the Orai3 channel can provide protection under hypoxic conditions. On the contrary, an increase in the Ca^2+^ response by 83% observed in the MDA-MB-231 cell line with increased Orai1 expression can reflect the accumulation of STIM under the membrane upon hypoxia induced by Na-Dit treatment. We can assume that hypoxia-induced acidification is not pronounced in MDA-MB-231 cells, which allows for the preservation of STIM–Orai coupling and the increase in SOCE.

Taken together, it could be concluded that BC cells with different organizations of SOCE could have not only quite different sensitivities to inhibitors, such as Leflunomid and Teriflunomid, but also exhibit different adaptations to external acidification and hypoxic conditions. So, more aggressive TNBC cells expose high SOCE at normal conditions, but they are quite well adapted to the acidic pH environment. On the other side, hypoxia increases SOCE in these cells even further, allowing TNBC to keep growing and spreading effectively even in hypoxic conditions. This would suggest that suppressing SOCE in triple-negative tumor cells could be effective in cancer treatment. However, in luminal A BC cells, SOCE is very moderate which would make using SOCE inhibitors ineffective. This should be taken into account when searching for an appropriate drug therapy.

## 5. Conclusions

Changes in the expression of STIM, Orai and TRPC proteins in breast cancer cell lines affect the amplitude and kinetics of the Ca^2+^ response caused by Ca^2+^ store depletion, which determines the ability of these cells to adapt to changes in the microenvironment, and, as a result, improve their survival. That is why the above proteins are potential targets for breast cancer therapy. We show that the potency of a novel Orai1 and Orai2 inhibitor, compound CM4620, is similar to that of the long-used BTP2 in the cell lines tested. In addition, the oxygen scavenger sodium dithionide demonstrates an inhibitory effect on the Orai3 channel in breast cancer cells with increased expression of this protein. Leflunomide and Teriflunomide inhibit SOCE in triple-negative breast cancer cells overexpressing Orai1, but not in cells overexpressing Orai3.

Thus, highly selective and general SOCE inhibitors are effective in all the cell lines tested, but probably have a low therapeutic potential due to their systemic effects on the body. On the other hand, less general SOCE blocking approaches show more selective effects on different subtypes of breast cancer cells.

## Figures and Tables

**Table 1:** Inhibitory analysis of SOCE in BC cell lines. Amplitude of Ca^2+^ response was measured at 9th minute after Tg application in the studied cell lines incubated with 5 µM CM4620, 5 µM BTP2 and 0.5% DMSO (control). Shown is the ratio of Fura-2 fluorescence at 340 and 380 nm, normalized to basal calcium levels, relative to control values in DMSO. The mean and standard error of the mean are shown; the significance level *p* is indicated in parentheses.

	DMSO	CM4620	BTP2	Lef	Ter
MCF-10A	100 ± 6.9%	11 ± 3% (*p* < 0.001)	8 ± 1% (*p* < 0.001)	66 ± 1% (*p* < 0.001)	88 ± 5% (*p* = 0.149)
MCF-7	100 ± 8.1%	32 ± 13% (*p* < 0.01)	35 ± 5% (*p* < 0.01)	104 ± 3% (*p* = 0.817)	102 ± 2% (*p* = 0.796)
MDA-MB-231	100 ± 3.7%	16 ± 1% (*p* < 0.001)	14 ± 1% (*p* < 0.001)	79 ± 2% (*p* < 0.001)	63 ± 1% (*p* < 0.001)

**Table 2:** Amplitude of the Ca^2+^ response at various extracellular pH and under hypoxic conditions. Amplitude of the calcium response at the 9th minute after Tg application relative to the basal Ca^2+^ level measured at various pH or after 1 mM Na-Dit incubation. Data were normalized to the SOCE amplitude at external pH 7.2. The mean and standard error of the mean are shown; the significance level *p* is indicated in parentheses.

	pH 6	pH 6.6	pH 7.2	pH 7.8	pH 8.4	pH 9	Na-Dit
MCF-10A	62 ± 6% (*p* < 0.001)	107 ± 7% (*p* = 0.712)	100 ± 3%	123 ± 7% (*p* < 0.01)	125 ± 7% (*p* < 0.01)	118 ± 4% (*p* < 0.001)	87 ± 2% (*p* < 0.01)
MCF-7	65 ± 3% (*p* < 0.001)	46 ± 8% (*p* < 0.001)	100 ± 2%	140 ± 3% (*p* < 0.001)	167 ± 7% (*p* < 0.001)	162 ± 7% (*p* < 0.001)	66 ± 5% (*p* < 0.001)
MDA-MB-231	32 ± 4% (*p* < 0.001)	11 ± 2% (*p* < 0.001)	100 ± 2%	209 ± 4% (*p* < 0.001)	255 ± 6% (*p* < 0.001)	307 ± 5% (*p* < 0.001)	183 ± 3% (*p* < 0.001)

**Table 3:** Efficacy of SOCE inhibition in BC cell lines. A decrease in the amplitude of the Ca^2+^ response as a percentage is shown at the 9th minute after applying Tg relative to control values. The mean and standard error of the mean are presented; the significance level *p* is indicated in parentheses. A dash indicates no inhibition.

	CM4620	BTP2	Lef	Ter	pH 6/6,6	Na-Dit
MCF-10A	89 ± 3% (*p* < 0.001)	92 ± 1% (*p* < 0.001)	34 ± 1% (*p* < 0.001)	12 ± 5% (*p* = 0.149)	38 ± 6% (*p* < 0.001)	13 ± 2% (*p* = 0.003)
MCF-7	66 ± 17% (*p* = 0.019)	57 ± 6% (*p* = 0.008)	-	-	54 ± 8% (*p* < 0.001)	34 ± 5% (*p* < 0.001)
MDA-MB-231	84 ± 1% (*p* < 0.001)	86 ± 1% (*p* < 0.001)	21 ± 2% (*p* < 0.001)	37 ± 1% (*p* < 0.001)	89 ± 2% (*p* < 0.001)	-

## Data Availability

Data are contained within the article.
